# Humanized yeast genetic interaction mapping predicts synthetic lethal interactions of FBXW7 in breast cancer

**DOI:** 10.1186/s12920-019-0554-z

**Published:** 2019-07-27

**Authors:** Morgan W. B. Kirzinger, Frederick S. Vizeacoumar, Bjorn Haave, Cristina Gonzalez-Lopez, Keith Bonham, Anthony Kusalik, Franco J. Vizeacoumar

**Affiliations:** 1 Department of Computer Science, College of Arts and Science, University of Saskatchewan, 176 Thorvaldson Bldg, 110 Science Place, Saskatoon, Saskatchewan S7N 5C9 Canada; 2 Department of Pathology and Laboratory Medicine, College of Medicine, University of Saskatchewan, 107 Wiggins Road, Saskatoon, Saskatchewan S7N 5E5 Canada; 3 Cancer Research, Saskatchewan Cancer Agency, 107 Wiggins Road, Saskatoon, Saskatchewan S7N 5E5 Canada; 4 Division of Oncology, College of Medicine, University of Saskatchewan, 107 Wiggins Road, Saskatoon, Saskatchewan S7N 5E5 Canada; 5 Cancer Cluster, Rm 4D01.5 Health Science Bldg, University of Saskatchewan, 107 Wiggins Road, Saskatoon, SK S7N 5E5 Canada

**Keywords:** Synthetic lethality, Breast cancer, Gene expression, Genetic interaction network

## Abstract

**Background:**

Synthetic lethal interactions (SLIs) that occur between gene pairs are exploited for cancer therapeutics. Studies in the model eukaryote yeast have identified ~ 550,000 negative genetic interactions that have been extensively studied, leading to characterization of novel pathways and gene functions. This resource can be used to predict SLIs that can be relevant to cancer therapeutics.

**Methods:**

We used patient data to identify genes that are down-regulated in breast cancer. InParanoid orthology mapping was performed to identify yeast orthologs of the down-regulated genes and predict their corresponding SLIs in humans. The predicted network graphs were drawn with Cytoscape. CancerRXgene database was used to predict drug response.

**Results:**

Harnessing the vast available knowledge of yeast genetics, we generated a Humanized Yeast Genetic Interaction Network (HYGIN) for 1009 human genes with 10,419 interactions. Through the addition of patient-data from The Cancer Genome Atlas (TCGA), we generated a breast cancer specific subnetwork. Specifically, by comparing 1009 genes in HYGIN to genes that were down-regulated in breast cancer, we identified 15 breast cancer genes with 130 potential SLIs. Interestingly, 32 of the 130 predicted SLIs occurred with *FBXW7*, a well-known tumor suppressor that functions as a substrate-recognition protein within a SKP/CUL1/F-Box ubiquitin ligase complex for proteasome degradation. Efforts to validate these SLIs using chemical genetic data predicted that patients with loss of *FBXW7* may respond to treatment with drugs like Selumitinib or Cabozantinib.

**Conclusions:**

This study provides a patient-data driven interpretation of yeast SLI data. HYGIN represents a novel strategy to uncover therapeutically relevant cancer drug targets and the yeast SLI data offers a major opportunity to mine these interactions.

**Electronic supplementary material:**

The online version of this article (10.1186/s12920-019-0554-z) contains supplementary material, which is available to authorized users.

## Background

Genetically, cancer is a complex disease with no two patients exhibiting the same tumor genetic profiles. Recent advances in tumor sequencing and identification of key driver genes has allowed the development of more targeted treatment strategies by leveraging individual patient genetics [1]. However, the druggability of these targets becomes challenging if these genes are not expressed or are down-regulated in cancers.

Synthetic lethality takes advantage of functional genetic interactions between gene pairs to develop targeted therapies and are beginning to be appreciated as a method of choice [2–4]. Inhibiting the synthetic lethal (SL) partner with a therapeutic drug selectively eliminates cancer cells leaving normal cells unaffected. Through the exploitation of these interactions, we can maximize the efficiency of personalized treatment and, ultimately, minimize the side effects that a patient experiences as a result of the therapeutic drug. Although the advent of genome-wide *sh*RNA and CRISPR screens have provided the specificity needed to perform the comprehensive epistasis mapping on any number of chosen gene pairs [5–7], experimentally testing all possible gene pairs across multiple cell types to identify SL interactions (SLIs) is laborious and time-consuming. As early as 1997 Hartwell et al. hypothesized the existence of conserved SLIs identified initially in yeast that could be used for therapeutic intervention in human cancers [8]. Subsequently, yeast genetic interaction data has been used to identify SLIs in humans [9–11]. These yeast-directed approaches have helped us to uncover several interactions in human cells [9–11]. For example, McManus et al. generated the first humanized SLIs of *RAD54B*-deficient human colorectal cancer cells by specifically translating the corresponding yeast interactions to human cancer cells [11]. Thus, rather than translating all the yeast interactions to human, contextualizing these interactions provided valuable insights.

Here we describe a patient-data driven approach where conserved SLIs of genes whose expression is lost in tumors alone are examined. Harnessing the vast available knowledge of yeast genetics and using yeast-human ortholog mapping, we generate a Humanized Yeast Genetic Interaction Network (HYGIN) that has the potential to identify novel cancer-specific SLIs and ultimately novel treatment strategies. This network is then integrated with patient data from The Cancer Genome Atlas (TCGA) (https://www.cancer.gov/tcga/) to identify genes that are down-regulated in breast cancer and provide a breast cancer-focused version of HYGIN. From this work, we predict novel SLIs that can be exploited for patient-specific cancer therapeutics. Some of these involve FBXW7, a well-known tumor suppressor.

## Methods

### Generating the humanized genetic interaction network

In order to generate HYGIN, experimentally validated yeast interactions from the May 2016 release of The Cell Map [12] were used. The yeast network contains quantitative genetic interactions for all gene pairs in *S. cerevisiae* and is the result of double mutant arrays conducted in yeast that represent all of the SL interactions in the yeast genome. The yeast interaction data contains ~ 550,000 negative genetic interactions (SL interactions) between ~ 90% of the genes in the yeast genome [12]. In order to generate a humanized network, the yeast network was translated in three stages: yeast gene name to yeast protein, yeast protein to human orthologous protein, and finally human protein to human gene name. The UniProt database [13] was used for the first two stages and InParanoid Version 8.0 [14], an online tool for identifying orthologs between two species, was used to identify the human orthologs to the yeast proteins in stage three. Strict one-to-one mapping with InParanoid was used for the network to prevent ambiguities in the translation process (Additional file 2: Table S1). The humanized gene interaction network and subnetworks were generated through a combination of bash (UNIX shell) and Python scripts, and imported into Cytoscape [15] for visualization and analysis. The resulting network has 1009 nodes (human genes) and 10,419 edges (proposed SL interactions between human genes). A summary of this process can be found in Fig. 2b.

### TCGA expression analysis

Gene expression data was downloaded from the TCGA database (https://www.cancer.gov/tcga/). Gene expression data for cancerous and breast normal tissue in the same patient was available for 114 patients: level-3 HiSeq RSEM gene-normalized RNA-seq data was obtained for 1104 cancerous and 114 normal samples for 20,530 genes. However, some genes had zero values for one or more patients as a result of either there being no transcripts, or transcripts missing for a particular gene. To avoid introducing uncertainty into the statistical analysis, only genes that had non-zero values for all 114 patients in both the cancerous tissue and the normal tissue were included. The data for these 13,983 genes was analyzed using the Shapiro-Wilk test for normality. It was found that data for 73% of the genes in the cancerous dataset, and 45% of the genes in the normal dataset had a normal distribution. As a result, the non-parametric Wilcoxon Signed Rank test was used on the paired data to determine if the median between the cancer sample and the normal sample was statistically different (*P* < 0.05). Since greater numbers of statistical inferences made from a dataset increases the chances of an error in any of those inferences, it was imperative to account for multiple hypotheses. The Benjamini-Hochberg procedure for multiple hypotheses comparison was used to adjust *P*-values. To determine the fold change of gene expression between the cancerous and normal tissue, the ratio of gene expression was then calculated (cancer/non-cancer) and the log-base-2 of the ratio was used as the fold change in gene expression in breast cancer. Significant genes were identified using a 2-fold cut off and statistically significant *P*-values (*P*-value after adjustment < 0.05).

### Generating the breast cancer specific sub-network

The breast cancer down-regulation sub-network was generated by extracting the genes that were down-regulated in breast cancer and all of their SL interactions from HYGIN. The result was a sub-network containing the potential 130 SL interactions that were specific to breast cancer between 130 genes in total, including the 15 genes that were down-regulated in breast cancer.

### Verifying overlap with previous work

In order to compare the overlap between HYGIN and the previous work from Deshpande et al. [9]*,* the latter’s network was filtered to remove any genes that had many-to-one, many-to-many, or one-to-many mappings, leaving only those with one-to-one mappings. As a result, the 10,419 interactions in HYGIN were compared to 7614 interactions in the Deshpande et al. network. Network comparison was completed using NetworkX in Python.

### Verifying one-to-one ortholog mapping of FBXW7

To confirm that there were no additional yeast-human orthologs of FBXW7, the InParanoid database was searched twice: the human genome was searched for the protein MET30 (the yeast protein that is orthologous to FBXW7 in humans) and CDC4 (a synonym for FBXW7 in humans).

### Gene expression in other cancers

The 15 genes that were identified to be down-regulated in breast cancer were also analyzed in the 24 types of cancer in the TCGA database. Gene expression for cancer tissue was compared to gene expression for normal tissue within each of the cancer types. Only the types of cancer where more than 7 of the 15 genes were identified to have lower expression in cancer than in normal tissue were included in the results.

### Drug data analysis

We performed a drug sensitivity assay using the data from the cancerRXgene database which contains drug response data for many cell lines [16]. The cell lines in the database were divided into two groups: those that have high expression of *FBXW7* and those with low. Briefly, *FBXW7* expression for each cell line was used to classify all the cell lines into two groups, high and low *FBXW7* expression, using the average *FBXW7* expression across all cell lines as a cut-off. Resulting drug response doses (IC_50_ values) were compared between the two groups. The IC_50_ values for each group were used to generate a *P*-value (Mann-Whitney U test). The survival percentage data from the cancerRXgene database for each drug at different concentrations was the basis for the dose-response curves.

## Results

### Construction of humanized yeast genetic interaction network (HYGIN) predicted 10,419 potential interactions involving 1009 human genes

**Fig. 1:**
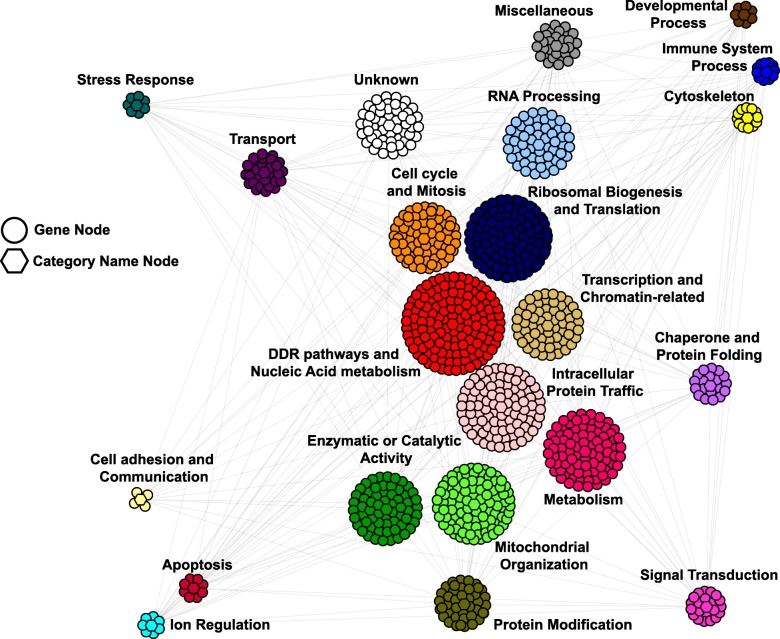
Network representation of HYGIN. Genes are grouped into clusters with an extra central node, which represents the category name (GO slim term; see Additional file 4: Table S3). Edges connect categories (clusters) only

Previously, a yeast genetic interaction network, The Cell Map, containing ~ 550,000 negative interactions has been described [12]. Using this information and a strict one-to-one ortholog mapping from yeast to human from InParanoid [14] (Additional file 2: Table S1), we generated a refined Humanized Yeast Genetic Interaction Network (HYGIN) (Fig. 1). HYGIN contains all of the yeast orthologs of human genes and their predicted SLIs. Of the ~ 550,000 negative genetic interactions in yeast (Costanzo et al.; http://thecellmap.org/costanzo2016/, updated May 2016) we evaluated only statistically significant interactions with a *P*-value < 0.05 that were also strong negative interactions where ε < − 0.2. Using InParanoid mapping from yeast to human, the yeast network was reduced to predictive negative interactions that exist between the human orthologs of these yeast genes. The resultant HYGIN contains 1009 human genes and 10,419 proposed SLIs (Additional file 3: Table S2). Topology of this network combined with our Gene Ontology slim terms (Additional file 4: Table S3) shows dense clustering of genes involved in DNA damage and repair pathways and cell cycle regulators, or RNA processing and ribosome biogenesis and translation components, suggesting we have recovered meaningful humanized genetic interactions as these are highly conserved processes across the evolutionary trajectory (Additional file 1: Figure S1). Since there are only 1266 yeast genes that map to exactly one human ortholog, it is not surprising that the total number of human genes in the humanized network is less than that in the starting yeast network, as only a fraction of these genes has unique human orthologs.

A similar approach to generate a list of potential human SLIs based on a previous version of the yeast dataset has been published by Deshpande et al. [9]. Although this previous work used the same cut-offs (*P* < 0.05 and ε < − 0.2), they also used a relaxed cut-off (ε < − 0.08) for SLIs that were identified in reciprocal screens. However, to maintain stringency in our work, we used a consistent cut-off value (ε < -0.2) irrespective of whether an interaction was identified in a reciprocal screen or not. This previous work was compared to HYGIN to determine if there were more interactions identified in our work. Compared to Deshpande et al. who identified 1522 potential SLIs in humans, we have identified 10,419 interactions; however, a total of 777 edges were in common between the two networks (Additional file 1: Figure S2, Additional file 5: Table S4) [9]. One of the reasons that HYGIN contains more SLIs is that our study uses a more recent version of The Cell Map database that includes interactions between essential and nonessential yeast genes.

### Identification of down-regulated genes in breast cancer and building a breast cancer-specific SL interaction network

**Fig. 2:**
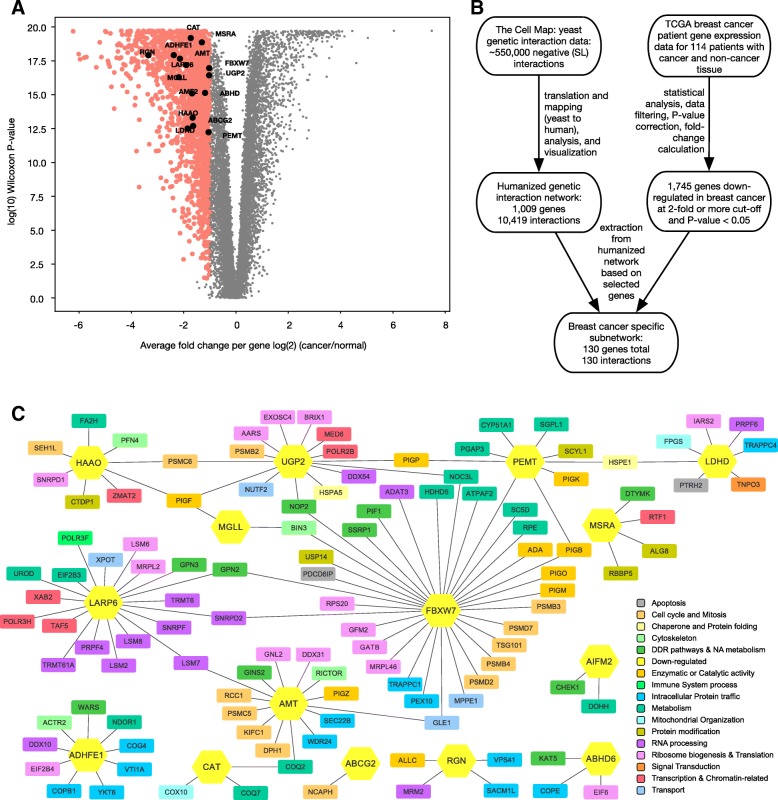
Breast cancer sub-network of SL interactions. **a** Volcano plot of the average change of expression per gene expressed as log_2_ (cancer/normal) plotted against the log_10_ of the adjusted Wilcoxon *P*-value. Dots in red represent genes that have an average 2-fold or more decrease in expression. Back dots represent the 15 genes that are also in the HYGIN network. **b** Flow diagram showing the flow of data and methods used to generate the breast cancer specific subnetwork. The final breast cancer network had 15 genes that are down-regulated in breast cancer and 115 genes that interact with them. As a result, there are 130 genes (nodes) total in the breast cancer subnetwork. **c** Visualization of the breast cancer specific subnetwork generated using 15 genes in HYGIN that were found to be down-regulated in breast cancer (yellow) and their corresponding SL gene pairs. Edges represent proposed SL interactions between genes, and nodes (coloured based on GO slim terms) represent SL partners of the 15 genes

To make HYGIN more applicable for human cancer therapeutics, we focused on breast cancer and developed a breast cancer-specific subnetwork. Exploiting any yeast SLI may not be beneficial if neither of the genes is altered in cancers. As a result, in order to identify those interactions in HYGIN that are relevant to breast cancer, we used TCGA data (https://www.cancer.gov/tcga/) to identify genes that are down-regulated in breast cancer. Although there are gene expression data for over 1000 breast cancer patients available in TCGA, because genes expression is a relative measurement, we chose to use only those tumor samples that had matching normal gene expression data. That is, we used data only from patients where gene expression results were available for both cancer and normal tissue. This stringent approach was followed to eliminate those genes that are down-regulated in cancer, but whose expression in normal tissue is also low. For each patient, gene expression data for cancerous and normal tissue was compared to generate a log-base-2 ratio of cancerous to normal gene expression. From this, we identified 1745 genes that were statistically down-regulated in breast cancer with at least a 2-fold change in expression cut off (*P* < 0.05) (Fig. 2a, Additional file 6: Table S5). Of these 1745 genes that are statistically down-regulated in breast cancer, 181 were previously identified in the Tumor Suppressor Gene database (TSG; https://bioinfo.uth.edu/TSGene/) as potential tumor suppressors.

The resultant gene set was compared to the HYGIN network, and it was found that 15 genes were both down-regulated in breast cancer and present in the HYGIN network. These 15 genes included some of the well-established tumor suppressors like *CAT* [17] and *FBXW7* [18]. Using a systematic effort (as depicted in the flow diagram in Fig. 2b), we generated the breast cancer specific subnetwork. This subnetwork shows these 15 genes and the 115 genes that they have SLIs with for a total of 130 genes (Fig. 2c, Additional file 7: Table S6). By targeting the SL partners of the 15 genes that are already down-regulated in breast cancer, novel targeted therapeutics can be developed to provide treatment options for patients.

Furthermore, the breast cancer specific subnetwork from this study was also compared to the 7614 interactions in the Deshpande et al. [9] network and 16 interactions were identified in both networks. As a result, 12% of the interactions in the breast cancer specific subnetwork from this study overlap with the previous work published by Deshpande et al. It is interesting to note that 6 of the interactions with *FBXW7* that are in the breast cancer specific subnetwork were also identified previously in the Deshpande et al. network. The 6 interactions are between *FBXW7* and each of *ADA*, *BIN3*, *PDCD6IP*, *PEMT*, *PEX10*, and *USP14*.

### Synthetic lethal interactions of FBXW7 and its validation

**Fig. 3:**
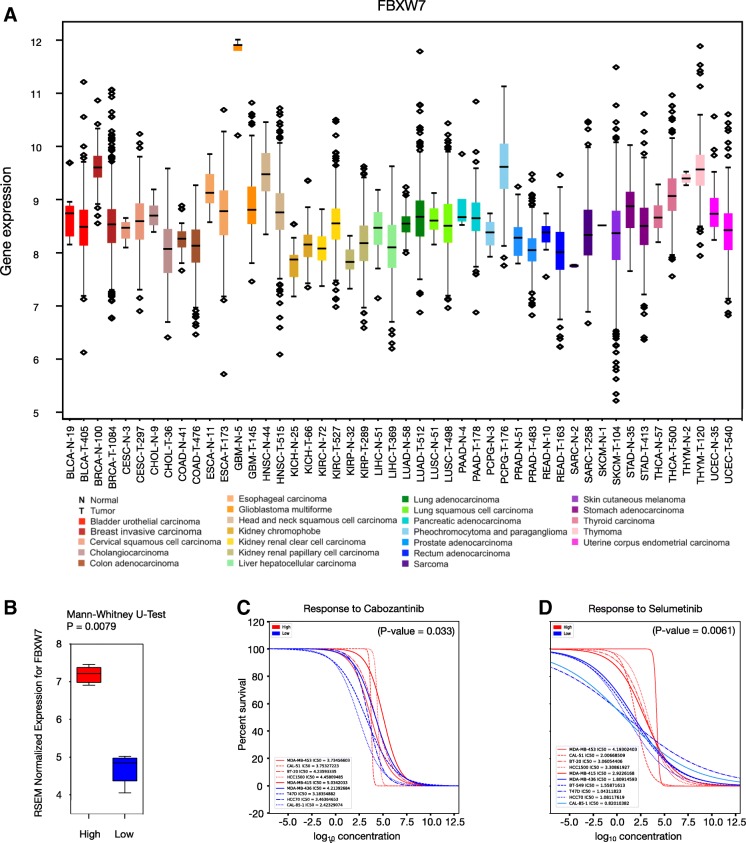
Analyses of SL interactions of *FBXW7*. **a** TCGA gene expression of *FBXW7* in 24 cancer types and corresponding normal tissue as calculated from RNA-seq data by expectation-maximization (log_2_). The averages of normal and tumor expression of *FBXW7* for the same tissue type are plotted beside each other and colour-coded based on cancer type. Numbers of patient samples are indicated in the x-axis labels. **b** Box plot classifying multiple cell lines from the cancerRXgene database based on the expression of *FBXW7*; up-regulated (red) and down-regulated (blue). **c**-**d** Drug kill curves for cell lines where *FBXW7* is down-regulated in blue, and cell lines that have up-regulated *FBXW7* in red. **c** Drug curves for Cabozantinib, and (**d**) Selumetinib

Having identified *FBXW7* as a candidate gene worth exploring further, its yeast ortholog and expression in other cancers was investigated. The yeast gene *MET30* encodes an F-box protein containing five copies of the WD40 motif and is known to control cell cycle function as part of a E3 ubiquitin ligase complex [19]. According to InParanoid, FBXW7 is the human ortholog of MET30 that shares similar roles in humans [14] (Additional file 1: Figure S3). In fact, FBXW7 is a well-known tumor suppressor that functions as a substrate-recognition protein within a SCF (SKP/CUL1/F-Box) E3 complex ubiquitin ligase complex, which targets numerous proteins for ubiquitin-mediated proteasomal degradation [20]. *FBXW7* is down-regulated not only in breast cancer, but also in 12 other cancers including colon, liver, lung, and prostate cancers (Fig. 3a, Additional file 1: Figure S4). However, *FBXW7* is also up-regulated in 6 other cancers including three types of kidney cancer, lung adenocarcinoma, and thyroid carcinoma (Fig. 3a, Additional file 1: Figure S4). Overall, our analysis identified 32 SL interactions *FBXW7*, many of which are proteasome components such as PSMB3, PSMB4, PSMD2, PSMD7, and USP14.

In order to validate the novelty associated with the identified SLIs in breast cancer, below we focus on two SLIs of *FBXW7* and provide evidence from drug screening data with mechanistic insight from literature. Our work predicted SLI between *FBXW7* and a member of the Pentose Phosphate Pathway (PPP), Ribulose-5-Phosphate-3-Epimerase (RPE), which catalyzes the reversible epimerization of D-ribulose 5-phosphate to D-xylulose 5-phosphate. Interestingly, previous work has shown that inhibition of the PPP results in decreased proliferation of tumor cell lines [21–25]. In addition, it has also been shown that the PPP is essential for metabolic network modulation to support tumor angiogenesis as inhibition of VEGFR-2 causes a decrease in PPP flux [26]. Thus, if inhibition of VEGFR-2 may decrease PPP flux, we hypothesized that inhibition of VEGFR should mimic the SL interaction between *FBXW7* and *RPE*. To test this idea, we used drug response data from the cancerRXgene database (https://www.cancerrxgene.org) and asked if cell lines deficient in *FBXW7* are sensitive to the VEGFR-2 inhibitor Cabozantinib. The cancerRXgene database contains data for 265 drugs and multiple cell lines and was examined to identify compounds that are more effective when used selectively with cell lines that have a low *FBXW7* expression. We found that cell lines with low expression of *FBXW7* were more sensitive to Cabozentanib (*P*-value = 0.033) (Fig. 3b, c), which supports our hypothesis that inhibition of VEGFR should mimic the SLI between *FBXW7* and *RPE*. Unfortunately, our classification of cell lines based on FBXW7 expression for only breast cancer cell lines did not yield sufficient sample size.

Similarly, we also found a SLI between *FBXW7* and *ADA*, an adenosine deaminase that regulates cellular levels of adenosine and deoxyadenosine. ADA, along with a few other enzymes (for example HGPRT), are responsible for purine metabolism and are well known targets for cancer chemotherapy [27]. ADA can both degrade adenosine and bind extracellularly to adenosine receptors to function as an allosteric modulator to regulate adenosine. In fact, studies in a human astrocytoma cells have shown that manipulation of cellular purine metabolite concentrations can make these cells more sensitive to apoptosis [28]. While it remains to be explored if the astrocytoma cell line is deficient in *FBXW7*, these studies indicate a potential role for *ADA* as a therapeutic target. Interestingly, previous studies have shown that the expression of *ADA* is induced by growth factors like IGF in a Ras-MAPK pathway dependent manner [29]. To test this idea, we used drug response data from cancerRXgene database and asked if cell lines deficient in *FBXW7* are sensitive to the *MEK* inhibitor Selumitinib that affects the Ras-MAPK pathway (Fig. 3d). Consistent with our SL prediction, we found cells with low expression of *FBXW7* to be more sensitive to Selumitinib (*P*-value = 0.0061). Thus, our predictive analyses indicate that *FBXW7* can be used as a biomarker to identify and treat patients with drugs like Selumitinib that indirectly affect the expression of *ADA*. Importantly, the availability of *ADA* inhibitors like 2’deoxycoformycin (Pentostatin) that are already in the clinics as standard chemotherapeutic agents for lymphoid malignancies [30] may also represent a potential option to test our prediction.

## Discussion

In this work we describe a novel approach to identifying potential SLIs in breast cancer that exploits yeast genetic networks in conjunction with strict ortholog mapping and patient gene expression data. Yeast is a common model organism and generating a human orthologous network from a yeast gene network provides a major framework to extend yeast genetic interaction data to humans. When generating HYGIN, strict one-to-one yeast-human ortholog mapping was used to avoid ambiguity as there are several instances where a single yeast protein map to multiple human orthologs, and vice versa. HYGIN is cancer independent, as no assumptions about cancer type are made while generating the network. As a result, any cancer gene expression data can be subsequently applied to generate cancer type specific subnetworks of genetic interactions. We generated a breast cancer specific subnetwork of HYGIN using breast cancer patient data from TCGA.

Most studies tend to evaluate additional data as a means of reaching statistically significant results, or more data as a means of completing an exhaustive analysis [31, 32]. However, we used a more stringent methodology and only analyzed TCGA patient data for which there was gene expression data for both breast cancer tissue and normal breast tissue. This not only increases the confidence in actual gene up- and down-regulation, but it also helps eliminate false positive results that occur due to differences in basal gene expression between individuals. By comparing up- and down-regulation of genes between two like-tissue samples from the same patient, changes in expression are more reliable than pooling cancer samples and comparing them to pooled normal samples. Although this strategy is very stringent, it is also one of the strengths of our gene expression analysis.

The breast cancer specific interaction network produced 130 SL interactions between 15 genes down-regulated in breast cancer and their 115 SL partners. The 15 genes that were identified to be down-regulated in breast cancer were also assessed in 24 other cancer types. Of the 15 genes, 11 of them were statistically down-regulated in more than 15 cancer types including breast cancer (Additional file 1: Figures S5, S6, S7 and S8). Of note, *HAAO*, *MGLL*, and *UPGE* were down-regulated in 18 cancer types; *ADHFE1*, *CAT*, and *MSRA* were down-regulated in 19; and *RGN* was down-regulated in 21 different types of cancer. This information suggests that SLIs identified in breast cancer for these genes would be extremely valuable to explore in other cancers as well. The subnetwork, and ultimately this subset of SLIs, are an ideal starting point for in vitro and in vivo validation studies and suggest novel targeted therapeutic strategies. By extracting 15 genes that were found to be down-regulated in humans and their SL partners, we can target their SL partners with known or novel drugs to develop new treatment strategies on a patient-by-patient basis. Moreover, knowledge in cancer genomics (and hence the literature) is not complete. Hence it is quite possible for a legitimate interaction to exist in HYGIN that is not (yet) documented in the literature. To determine any of these as false positives is difficult; it would require literature that explicitly rules out the interactions from ever actually occurring. Further, we note that the interactions reported by HYGIN that are also not described in the literature are one of the main contributions of HYGIN. Therefore, we feel that HYGIN provides a promising list of potential candidates for future work.

Overall, our analysis identified 32 SL interactions *FBXW7*, many of which are proteasome components such as PSMB3, PSMB4, PSMD2, PSMD7, and USP14. Given that FBXW7 is a E3 ubiquitin ligase, these interactions highlight the genetic property of SL relationships where functional coherence is often observed. As cyclin E is a substrate of proteasome degradation, it is interesting to note that the recently published SLI between *FBXW7* and *CCNE1* may reflect the interaction between FBXW7 and proteasome components [33]. Our stringent ortholog mapping is one-to-one and unfortunately, cyclin E falls into the “more than one ortholog” category in yeast and “more than one corresponding ortholog” in humans, and as a result was not included in HYGIN.

Similarly, our computational prediction suggested a SLI between *FBXW7* and *USP14*, a gene that codes for a deubiquitinating enzyme. FBXW7 has been proposed in the degradation of a number of substrates including Aurora B [34, 35]. Although Aurora B kinase is primarily degraded through the Anaphase-Promoting Complex/Cyclosome (APC/c) [36], negative regulation of Aurora B by FBXW7 plays an important role in Aurora B ubiquitination and degradation [35]. USP14 is a major regulator of the proteasome and one of three proteasome-associated deubiquitinating enzymes, which also affects protein turnover in a substrate-specific manner [37]. Interestingly, a recent study has reported that over-expression of *USP14* stabilized and prevented Aurora B degradation through deubiquitination [38]. Furthermore, a FBXW7-Aurora B-p53 negative feedback loop has also been suggested [39]. This feedback loop suggests that a loss of *FBXW7* leads to an increase in Aurora B, which phosphorylates p53 and leads to MDM2 enhanced degradation of p53 and ultimately cancer cell growth [39]. While the loss of FBXW7 may already stabilize Aurora B [34, 35], over-expression of *USP14* may be necessary for continued stability of Aurora B to maintain cell-cycle progression and cell survival. Thus, consistent with our observation, loss of USP14 when *FBXW7* is down-regulated may destabilize Aurora B, leading to a SL phenotype. This meaningful interpretation of the SLIs between FBXW7 and aurora kinases or proteasome components reiterate the potential opportunity of exploiting yeast genetics and the power of HYGIN to identify and predict mechanistically relevant relationships between gene pairs.

Although we have derived supporting data for these SLIs from either drug response data from the cancerRXgene database (https://www.cancerrxgene.org) or from previous publications, we acknowledge that our predictions require further experimental validations in cell line or animal models of breast cancer. Moreover, SLIs of FBXW7 are potential targets for cancer therapeutics in only some cancers as not all cancers have decreased expression of FBXW7. For example, FBXW7 is also up-regulated in 6 other cancers including three types of kidney cancer, lung adenocarcinoma, and thyroid carcinoma (Fig. 3a, Additional file 1: Figure S4). In these instances, the SLIs we have identified will not work. While there are several genes that are either up- or down-regulated in multiple cancers, in the context of synthetic lethality, only those patients with low expression of FBXW7 will benefit from this therapy [40]. Therefore, it would not be appropriate to use this strategy on all tumor types. While targeting SLIs of *FBXW7* may have a wide opportunity for clinical application, it must be used in conjunction with genetic testing and patient gene expression data. Another caveat in our study is that not all classical breast cancer-related tumor suppressors have yeast orthologs. And even if they do, if any of them become an essential gene in yeast, then the current yeast data does not capture this efficiently. Although temperature-sensitive (ts) mutants are used for capturing genetic interactions, distinct ts mutants capture distinct genetic interactions. Thus, as it stands, for the 1745 genes that are down-regulated in breast cancer, even though 181 were previously identified in the Tumor Suppressor Gene database (TSG; https://bioinfo.uth.edu/TSGene/), only 15 of these had yeast orthologs that had interactions in HYGIN. Thus, as powerful as it is, the evolutionary distance between yeast and human has its limitations.

Of note, *FBXW7* and *PEMT* are both down-regulated in breast cancer and they share a proposed SLI in HYGIN. The gene expression ratios for all 114 patients for both *FBXW7* and *PEMT* were evaluated to further investigate this finding. Results showed that 20% of patients have gene expression ratios less than 2-fold for both *FBXW7* and *PEMT*, and one patient had gene expression less than 4-fold for both genes. There are two possibilities that could lead to this result. First, it is possible that at some point in evolutionary time this SLI was valid. However, the cancer cells overcame the SL dependency of these two genes in humans, and what we expect to manifest as cell death results in cell survival. Second, it is possible that this result is a false positive. Previous work has shown that there is only 23% overlap in SLIs between two species of yeast (*S. cereviseae* and *S. pombe*) [41]. This means that although 65% of genes between these two species retain essential function, SL relationships are lost over evolutionary time. Thus, we are expecting to see a loss of some SLIs in our network as a result of a larger species gap between *Homo sapiens* and *S. cerevisiae*. Ultimately, this result highlights the need for careful biological testing of these SLIs. Though the network is based on valid research methods, cell lines and animal models behave differently than predicted results. Therefore, the breast cancer specific network contains interactions that are valuable starting points for SL research moving forward and provides a valuable source of SLIs for the scientific community to explore in vitro.

## Conclusion

Overall, this study provides a patient-data driven interpretation of yeast SLIs. We believe further extrapolation of the cancer-independent HYGIN network represents a novel strategy to derive therapeutically relevant cancer drug targets. Specifically, we present evidence for the identification of breast cancer-specific SLIs for the tumor suppressor FBXW7.

## Additional files

Additional file 1: **Figure S1.** Twenty-two GO Slim terms and the interactions between them are represented in this table. The interactions were translated from yeast. Cells are colour-coded according to the number of interactions between two groups of GO terms, where the dark red cells represent more interactions and white cells represent fewer interactions. **Figure S2.** Schematic showing overlap between this work and the work of Deshpande et al. [9]. The interactions in the two networks generated in this work (HYGIN and the breast cancer specific subnetwork) are compared to the “Complete Set” from the Deshpande et al. research. **Figure S3.** FBXW7 Orthologs. Two searches of the InParanoid database were conducted to verify that FBXW7 has a strict one-to-one mapping. A) The human genome was searched for the protein MET30 (the yeast protein that is orthologous to FBXW7 in humans). As expected, a single ortholog mapping was identified between *Saccharomyces cerevisiae* and *Homo sapiens*. B) The protein CDC4, a synonym for FBXW7, in humans was subsequently searched for in humans. Finding no ortholog in human, InParanoid reported “hits” in other organisms. Results showed that there is no entry for CDC4 in humans, which means that there is no additional FBXW7 ortholog in *Saccharomyces cerevisiae*. **Figure S4.** *P*-values for gene up- and down-regulation of the 15 genes found to be down-regulated in breast cancer. The 15 genes that are the basis for the breast cancer subnetwork were analyzed across 24 different cancer types. The 19 cancer types where the majority of the 15 genes are down-regulated are depicted here. Significant values in blue denote statistically significant lower expression in pooled cancer tissue when compared to pooled normal tissue, values in red denote statistically higher expression in cancer, and NS values denote a non-significant change in gene expression between cancer and normal tissues. All *P*-values were calculated using the Mann-Whitney U test. **Figure S5.** Gene expression calculated by RNA-seq by expectation-maximization (log_2_) for *ABCG2*, *ABHD6*, *ADHFE1*, and *AIFM2* for 24 different cancer types. Box plots are colour-coded in pairs based on cancer type where the boxplot on the left is gene expression in cancer and the plot on the right is gene expression in normal tissue. Labels on the x-axis represent the abbreviation of the cancer type, whether the sample represents tumor (T) or normal (N) tissue, and the sample size. **Figure S6.** Same as description as in Additional file 1: Figure S5, except for *AMT*, *CAT*, *HAAO*, and *LARP6*. **Figure S7.** Same as description as in Additional file 1: Figure S5, except for *LDHD, MGLL, MSRA, and PEMT.* **Figure S8.** Same as description as in Additional file 1: Figure S5, except for *RGN and UPG2. (PDF 5032 kb)*

Additional file 2: **Table S1.** Strict one-to-one ortholog mapping for yeast genes in humans. Ortholog mapping information was taken from InParanoid and filtered to identify instances where one yeast protein mapped to one human protein. The resulting 1,266 pairs of orthologs can be found in this table*. (XLS 134 kb)*

Additional file 3: **Table S2.** HYGIN network table. This table contains the initial mapping data from the yeast network where SL interactions are represented in pairs by A and B (Yeast Gene ID A, Yeast Gene ID B, Yeast Gene Name A, Yeast Gene Name B, *E*-value, and *P*-value). Through the 3-stage mapping process used to generate HYGIN, it also contains columns corresponding to Yeast Protein Accession (A and B), Human Protein Accession (A and B), and Human Gene Name (A and B). Human gene names were used to generate the network in Cytoscape. Due to some reciprocal screens in the initial yeast screen, there were some duplicate (reciprocal) entries in this table. Once the network was loaded into Cytoscape, “Remove duplicate edges” was used to eliminate reciprocal hits. This resulted in the 1,009 genes and 10,419 edges in the final HYGIN network. (XLS 2700 kb)

Additional file 4: **Table S3.** GO Slim mapping table. Twenty-two GO Slims used for colouring the HYGIN network in Cytoscape, courtesy of the Vizeacoumar research group. The common name for the human protein and its accession number accompany the GO Slim terms that have been curated by the Vizeacoumar research group. *DDR Pathways = “DNA Damage and Repair Pathways” and NA Metabolism = “Nucleic Acid Metabolism”. (XLS 141 kb)

Additional file 5: **Table S4.** Synthetic lethal interactions in common with previous work. List of the 777 SL interactions that are in common between HYGIN and the network published by Deshpance et al. [9]. GeneA and GeneB have an SL interaction according to both networks. (XLS 100 kb)

Additional file 6: **Table S5.** List of genes that were identified as down-regulated in breast cancer at a 2-fold cut-off and with a Wilcoxon Signed Rank *P*-value less than 0.05. Mean expression is the average of the log_2_ ratio (cancer gene expression/normal gene expression) for each gene. *P*-values are from Wilcoxon Signed Rank test (Python) after adjustment (Benjamini-Hochberg procedure), and log_10_(*P*-values) were used to generate the volcano plot in Fig. 2a. (XLS 230 kb)

Additional file 7: **Table S6.** Genetic interactions in the breast cancer subnetwork. The table contains a list of the SL gene pairs that make up the breast cancer specific subnetwork where column GeneA represents the genes that are down-regulated in breast cancer, and column GeneB are their SL gene pairs. (XLS 66 kb)

## Data Availability

Public data was used to analyze and generate the results. All supporting data are included within the article and its supplementary files.

## Abbreviations

HYGIN: Humanized Yeast Genetic Interaction Network
SL: Synthetic lethal
SLI: Synthetic lethal interaction
TCGA: The Cancer Genome Atlas
