# Supplementary material for: Humanized yeast genetic interaction mapping predicts synthetic lethal interactions of FBXW7 in breast cancer
Source: BMC Med Genomics. 2019 Jul 27;12:112. doi: 10.1186/s12920-019-0554-z (PMC6660958; doi:10.1186/s12920-019-0554-z)

Supplementary Figure S1

	Apoptosis	Cell Cycle and Mitosis	Cell adhesion and communication	Chaperone and Protein Folding	Cytoskeleton	DDR pathways and NA metabolism	Developmental Process	Enzymatic or Catalytic Activity	Immune System process	Intracellular Protein Traffic	Ion Regulation	Metabolism	Miscellaneous	Mitochondrial Organization	Protein Modification	RNA Processing	Ribosomal Biogenesis and Translation	Signal transduction	Stress Response	Transcription and Chromatin-related	Transport
Cell Cycle and Mitosis	18																				
Cell adhesion and communication	0	5																			
Chaperone and Protein Folding	12	61	0																		
Cytoskeleton	3	17	0	56																	
DDR pathways and NA metabolism	32	372	2	67	14																
Developmental Process	0	6	0	1	1	17															
Enzymatic or Catalytic Activity	9	49	2	25	23	94	3														
Immune System process	0	19	0	7	0	18	0	5													
Intracellular Protein Traffic	26	176	4	77	33	245	14	225	20												
Ion Regulation	1	12	0	2	1	13	1	1	2	5											
Metabolism	8	89	1	41	23	135	8	97	20	201	6										
Miscellaneous	6	69	1	18	10	60	0	46	4	104	1	36									
Mitochondrial Organization	13	52	2	27	13	123	8	52	9	101	11	83	23								
Protein Modification	6	62	3	16	6	57	2	38	8	114	2	48	26	36							
RNA Processing	17	117	4	36	19	201	12	49	30	126	3	78	45	53	37						
Ribosomal Biogenesis and Translation	21	197	7	29	36	248	13	78	22	173	8	144	81	102	58	218					
Signal transduction	6	22	1	21	4	32	6	29	2	56	1	34	14	13	11	31	47				
Stress Response	0	11	0	5	2	22	1	2	1	6	0	4	3	4	4	7	10	0			
Transcription and Chromatin-related	20	157	3	42	23	163	4	46	23	149	9	106	34	59	61	120	173	23	4		
Transport	6	55	1	4	6	53	3	32	3	72	3	37	9	25	18	26	55	8	1	34	
Unknown	5	45	3	29	9	75	5	22	5	105	4	45	23	37	22	64	84	20	1	55	16

Supplementary Figure S2

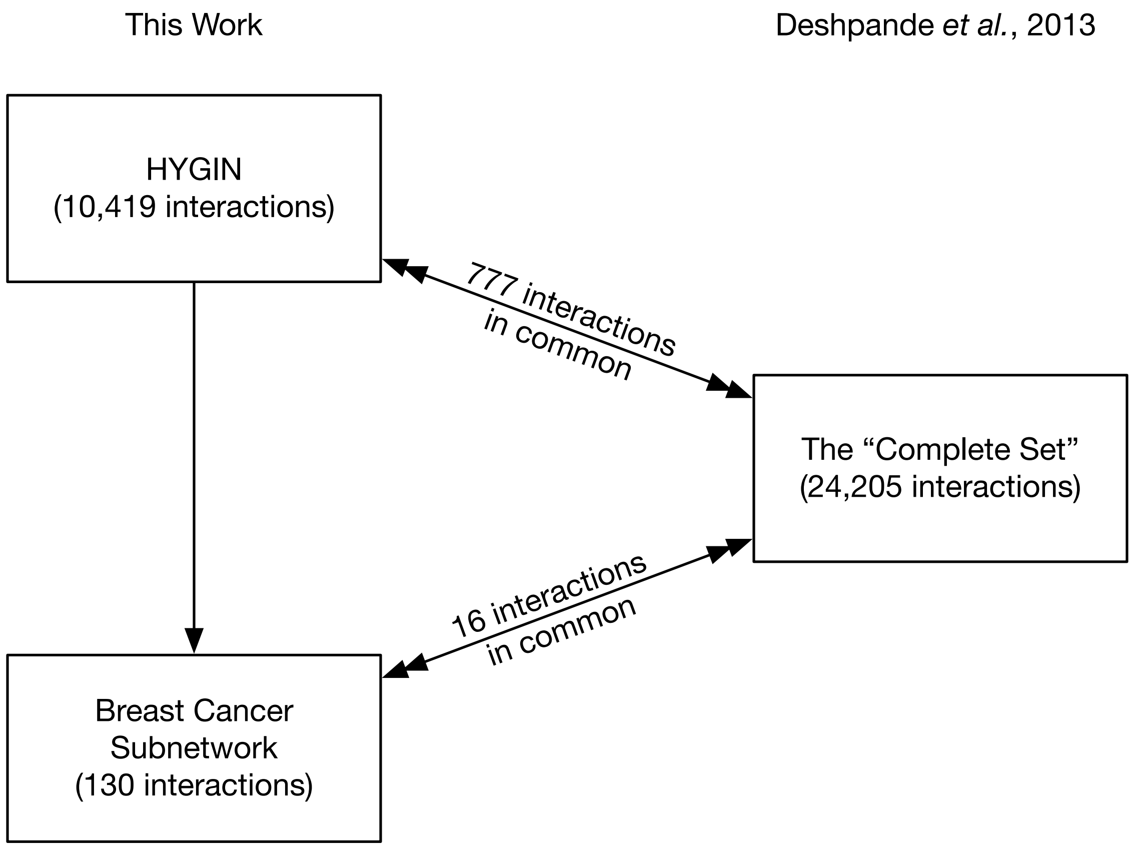

Supplementary Figure S3

A

Searching the species
Homo sapiens
for the identifier **met30** excluding inparaloga scoring below 0.05

xml

Inparalog and Orthologs cluster for Saccharomyces cerevisiae and Homo sapiens

Cluster 817					
**Protein ID**	**Species**	**Score**	**Bootstrap**	**Description**	**Alternative ID**
P39014	Saccharomyces cerevisiae	1	43%	F-box protein MET30	MET30_YEAST (UniProt)
Q969H0	Homo sapiens	1	99%	F-box/WD repeat-containing protein 7	FBXW7_HUMAN (UniProt)

B

Searching the species
Homo sapiens
for the identifier **cdc4** excluding inparalogs scoring below 0.05
More than one protein found:

Inparanoid	Gene ID	Protein ID	Species	Description	Alternative ID
Cluster	CDC4	A3LV96	Scheffersomyces stipitis	F box protein, for ubiquitin-dependent degradation	A3LV96_PICST (UniProt)
Cluster	CDC4	Q5A9A6	Candida albicans	Potential ubiquitin ligase F-box subunit	Q5A9A6_CANAL (UniProt)

Supplementary Figure S4

Gene Name	Bladder urothelial carcinoma	Breast invasive carcinoma	Cholangiocarcinoma	Colon adenocarcinoma	Esophageal carcinoma	Glioblastoma multiforme	Head and neck squamous cell carcinoma	Kidney chromophobe	Kidney renal clear cell carcinoma	Kidney renal papillary cell carcinoma	Liver hepatocellular carcinoma	Lund adenocarcinoma	Lung squamous cell carcinoma	Pheochromocytoma anc paraganglioma	Prostate adenocarcinoma	Rectum adenocarcinoma	Stomach adenocarcinoma	Thyroid carcinoma	Uterine corpus endometrial carcinoma
ABCG2	5.88E-05	2.48E-46	2.15E-06	2.06E-25	NS	NS	5.88E-04	4.82E-02	9.48E-07	7.70E-15	5.47E-12	4.36E-31	4.88E-24	2.22E-02	1.14E-11	2.28E-07	1.48E-03	7.29E-04	9.46E-20
ABHD6	3.12E-04	3.84E-39	3.22E-06	4.48E-22	6.29E-04	1.02E-03	NS	6.60E-06	NS	NS	6.36E-21	7.45E-30	6.47E-22	1.07E-02	2.57E-18	1.55E-05	1.24E-10	7.18E-11	2.16E-09
ADHFE1	8.84E-08	1.06E-54	2.15E-06	3.44E-25	1.15E-06	9.57E-04	3.12E-09	4.57E-06	4.22E-26	NS	5.55E-14	9.48E-14	2.31E-17	5.58E-03	NS	2.93E-07	3.84E-16	2.33E-14	6.87E-17
AIFM2	NS	1.47E-30	NS	9.61E-03	NS	NS	NS	3.22E-10	7.76E-05	2.87E-02	1.03E-12	6.83E-13	NS	1.10E-02	2.68E-15	NS	6.47E-03	1.00E-12	4.49E-12
AMT	5.01E-04	1.90E-34	2.15E-06	6.14E-15	1.74E-05	NS	NS	2.21E-07	6.21E-29	4.58E-08	4.05E-12	NS	4.26E-11	NS	4.87E-12	1.67E-02	3.73E-10	4.75E-02	2.00E-03
CAT	2.20E-05	1.16E-60	2.15E-06	8.34E-19	1.71E-04	1.17E-02	3.51E-07	NS	8.33E-34	1.28E-16	9.87E-25	1.29E-35	5.58E-32	1.66E-03	2.19E-09	6.83E-05	1.13E-08	2.62E-02	4.11E-07
FBXW7	2.37E-02	3.48E-57	4.50E-04	2.17E-02	4.87E-03	8.77E-05	1.56E-12	4.58E-05	4.88E-20	7.95E-06	1.09E-05	1.60E-02	4.53E-02	2.59E-03	3.69E-06	1.38E-02	3.09E-04	1.08E-12	8.50E-07
HAAO	6.77E-12	3.16E-40	2.15E-06	1.74E-03	NS	5.75E-03	1.08E-08	6.29E-10	9.83E-06	9.35E-03	3.65E-19	1.90E-09	1.50E-09	3.38E-02	1.21E-14	NS	1.94E-03	6.98E-04	2.63E-17
LARP6	8.70E-05	2.46E-53	3.56E-05	NS	1.88E-03	9.77E-05	6.33E-04	3.42E-06	3.86E-11	1.74E-02	3.96E-03	1.13E-07	3.13E-10	2.68E-03	8.67E-04	NS	2.78E-07	NS	5.28E-11
LDHD	NS	7.42E-31	2.15E-06	1.37E-25	4.23E-05	9.77E-05	3.86E-19	1.09E-07	6.10E-31	9.57E-04	6.55E-23	1.14E-05	9.66E-13	1.13E-02	NS	4.31E-07	2.96E-10	4.94E-17	NS
MGLL	4.90E-11	6.02E-46	2.05E-02	8.72E-24	4.79E-02	1.37E-03	1.33E-22	NS	2.57E-21	3.27E-02	2.38E-12	1.53E-15	2.31E-31	2.99E-02	3.74E-05	1.55E-05	9.14E-07	1.45E-02	5.21E-07
MSRA	7.13E-05	1.91E-49	2.15E-06	4.22E-14	5.80E-05	2.11E-02	2.15E-17	8.65E-09	7.74E-08	4.80E-09	1.68E-22	4.08E-19	1.34E-30	1.71E-02	6.22E-06	3.87E-04	2.55E-10	7.84E-12	3.88E-02
PEMT	5.19E-05	8.23E-20	2.15E-06	2.19E-15	1.92E-02	9.00E-04	2.92E-02	9.87E-04	2.86E-02	1.47E-06	3.51E-22	4.40E-02	NS	NS	4.59E-03	4.77E-05	NS	3.71E-15	2.11E-03
RGN	1.61E-09	4.13E-52	2.15E-06	2.35E-06	7.82E-06	NS	2.07E-14	7.61E-03	4.79E-19	8.57E-10	3.80E-19	4.48E-17	1.02E-30	1.61E-03	4.03E-15	1.57E-03	3.31E-10	4.50E-11	1.32E-16
UGP2	2.17E-05	1.36E-46	2.15E-06	2.41E-26	1.14E-04	1.58E-02	6.27E-16	2.62E-03	4.67E-15	6.75E-05	3.98E-20	4.26E-05	NS	1.50E-03	1.49E-09	1.31E-07	4.34E-08	5.93E-08	1.22E-05

Supplementary Figure S5

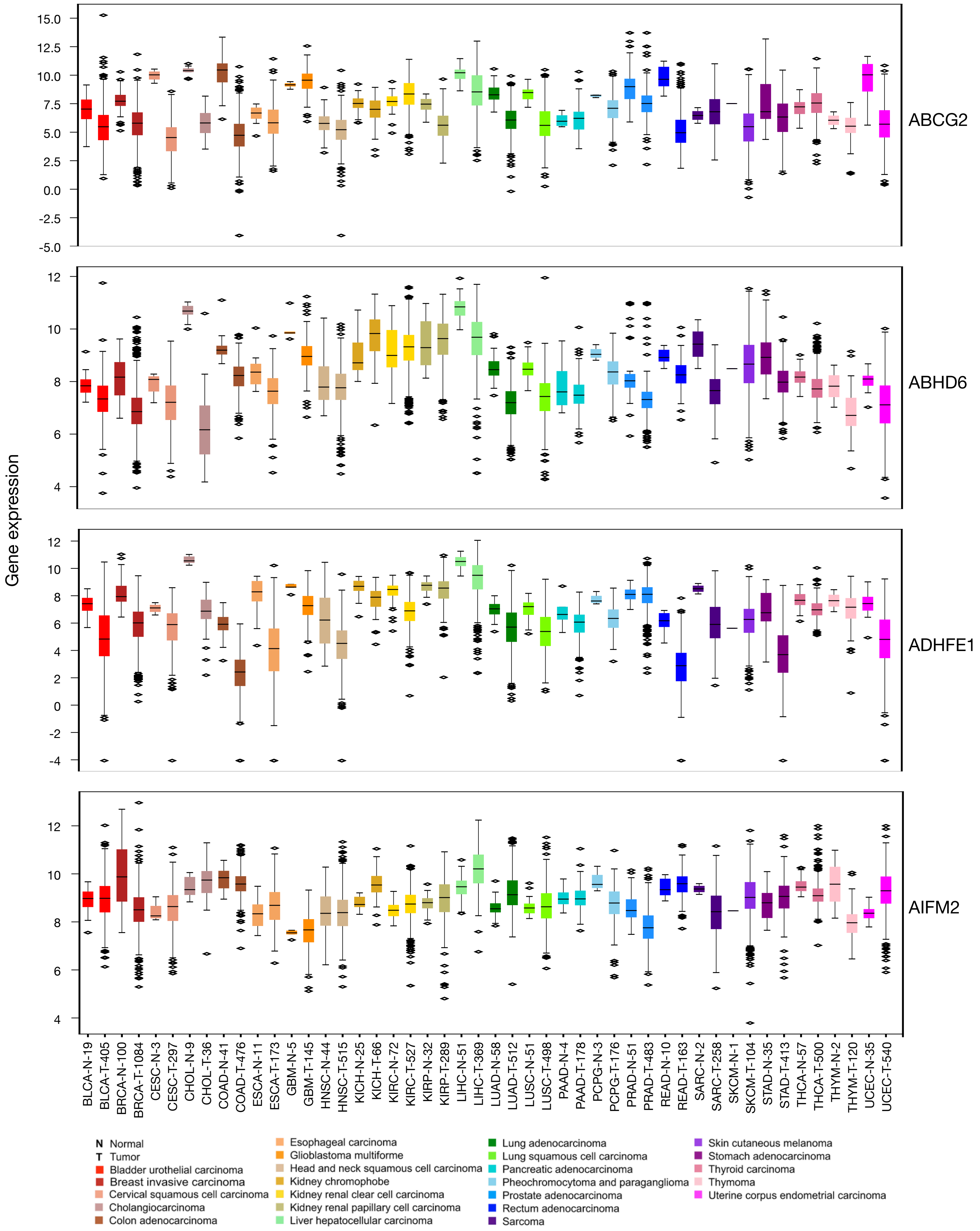

Supplementary Figure S6

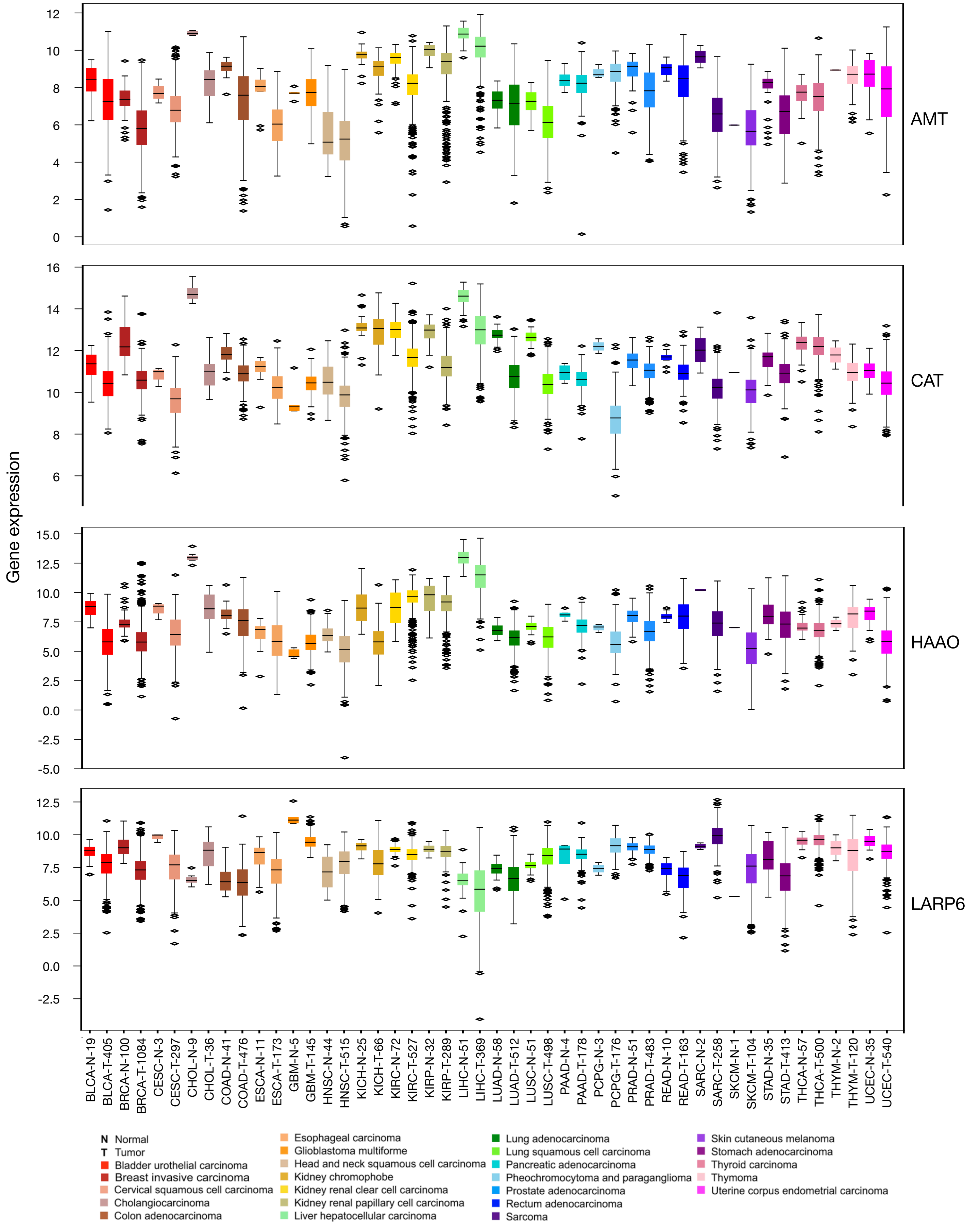

Supplementary Figure S7

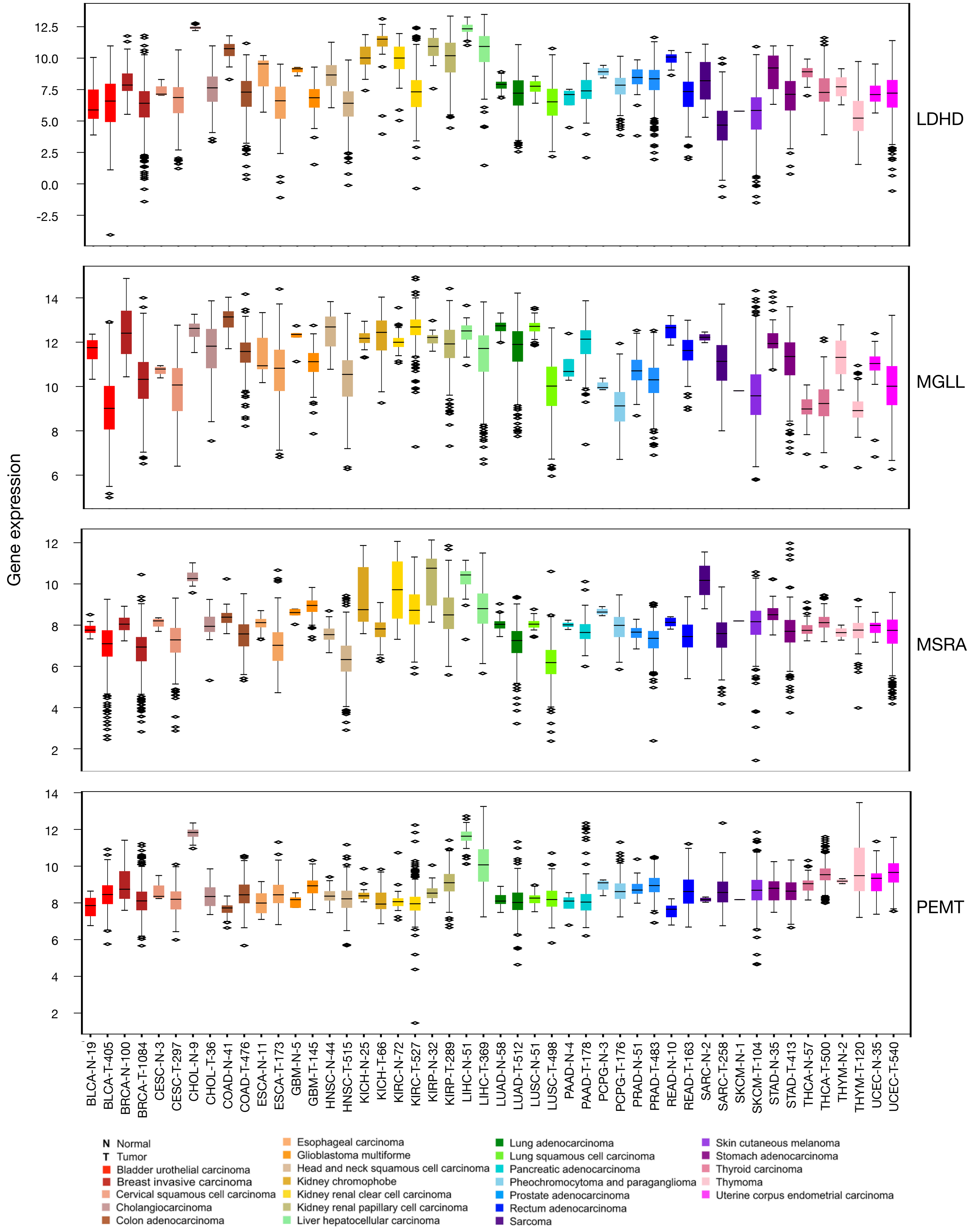

Supplementary Figure S8

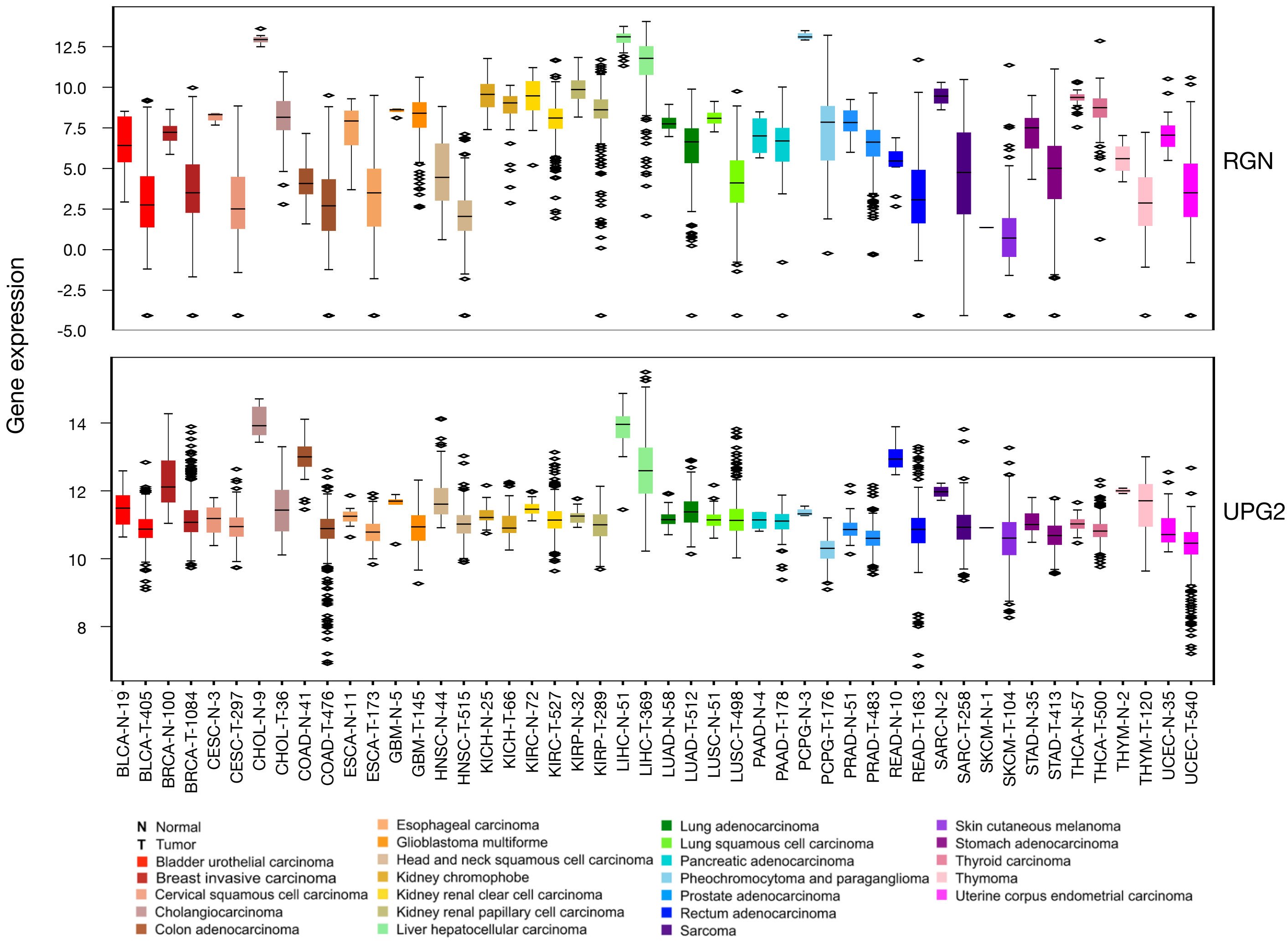

Supplement: Supplementary file 1 — Figure S1. Twenty-two GO Slim terms and the interactions between them are represented in this table. The interactions were translated from yeast. Cells are colour-coded according to the number of interactions between two groups of GO terms, where the dark red cells represent more interactions and white cells represent fewer interactions. Figure S2. Schematic showing overlap between this work and the work of Deshpande et al. [9]. The interactions in the two networks generated in this work (HYGIN and the breast cancer specific subnetwork) are compared to the “Complete Set” from the Deshpande et al. research. Figure S3. FBXW7 Orthologs. Two searches of the InParanoid database were conducted to verify that FBXW7 has a strict one-to-one mapping. A) The human genome was searched for the protein MET30 (the yeast protein that is orthologous to FBXW7 in humans). As expected, a single ortholog mapping was identified between Saccharomyces cerevisiae and Homo sapiens. B) The protein CDC4, a synonym for FBXW7, in humans was subsequently searched for in humans. Finding no ortholog in human, InParanoid reported “hits” in other organisms. Results showed that there is no entry for CDC4 in humans, which means that there is no additional FBXW7 ortholog in Saccharomyces cerevisiae. Figure S4. P-values for gene up- and down-regulation of the 15 genes found to be down-regulated in breast cancer. The 15 genes that are the basis for the breast cancer subnetwork were analyzed across 24 different cancer types. The 19 cancer types where the majority of the 15 genes are down-regulated are depicted here. Significant values in blue denote statistically significant lower expression in pooled cancer tissue when compared to pooled normal tissue, values in red denote statistically higher expression in cancer, and NS values denote a non-significant change in gene expression between cancer and normal tissues. All P-values were calculated using the Mann-Whitney U test. Figure S5. Gene expression calcul [file 12920_2019_554_MOESM1_ESM.pdf]
